# Improving Diabetes care through Examining, Advising, and prescribing (IDEA): protocol for a theory-based cluster randomised controlled trial of a multiple behaviour change intervention aimed at primary healthcare professionals

**DOI:** 10.1186/1748-5908-9-61

**Published:** 2014-05-24

**Authors:** Justin Presseau, Gillian Hawthorne, Falko F Sniehotta, Nick Steen, Jill J Francis, Marie Johnston, Joan Mackintosh, Jeremy M Grimshaw, Eileen Kaner, Marko Elovainio, Mark Deverill, Tom Coulthard, Heather Brown, Margaret Hunter, Martin P Eccles

**Affiliations:** 1 Institute of Health and Society, Newcastle University, Baddiley-Clark Building, Richardson Road, Newcastle Upon Tyne NE2 4AX, England; 2 School of Health Sciences, City University London, Northampton Square, London EC1V 0HB, UK; 3 Institute of Applied Health Sciences, University of Aberdeen, 2nd floor, Health Sciences Building, Foresterhill, Aberdeen AB25 2ZD, UK; 4 Department of Medicine, University of Ottawa, 451 Smyth Rd., Ottawa, Ontario K1H 8M5, Canada; 5 Ottawa Hospital Research Institute, The Ottawa Hospital - General Campus, 501 Smyth Road, Box 711, Ottawa K1H 8L6, Ontario, Canada; 6 National Institute for Health and Welfare, P.O. Box 30, Helsinki 00271, Finland; 7 Benfield Park Healthcare and Diagnostic Centre, Benfield Road, Newcastle upon Tyne NE6 4QD, UK

## Abstract

**Background:**

New clinical research findings may require clinicians to change their behaviour to provide high-quality care to people with type 2 diabetes, likely requiring them to change multiple different clinical behaviours. The present study builds on findings from a UK-wide study of theory-based behavioural and organisational factors associated with prescribing, advising, and examining consistent with high-quality diabetes care.

**Aim:**

To develop and evaluate the effectiveness and cost of an intervention to improve multiple behaviours in clinicians involved in delivering high-quality care for type 2 diabetes.

**Design/methods:**

We will conduct a two-armed cluster randomised controlled trial in 44 general practices in the North East of England to evaluate a theory-based behaviour change intervention. We will target improvement in six underperformed clinical behaviours highlighted in quality standards for type 2 diabetes: prescribing for hypertension; prescribing for glycaemic control; providing physical activity advice; providing nutrition advice; providing on-going education; and ensuring that feet have been examined. The primary outcome will be the proportion of patients appropriately prescribed and examined (using anonymised computer records), and advised (using anonymous patient surveys) at 12 months. We will use behaviour change techniques targeting motivational, volitional, and impulsive factors that we have previously demonstrated to be predictive of multiple health professional behaviours involved in high-quality type 2 diabetes care. We will also investigate whether the intervention was delivered as designed (fidelity) by coding audiotaped workshops and interventionist delivery reports, and operated as hypothesised (process evaluation) by analysing responses to theory-based postal questionnaires. In addition, we will conduct post-trial qualitative interviews with practice teams to further inform the process evaluation, and a post-trial economic analysis to estimate the costs of the intervention and cost of service use.

**Discussion:**

Consistent with UK Medical Research Council guidance and building on previous development research, this pragmatic cluster randomised trial will evaluate the effectiveness of a theory-based complex intervention focusing on changing multiple clinical behaviours to improve quality of diabetes care.

**Trial registration:**

ISRCTN66498413.

## Background

Type 2 diabetes is an increasingly prevalent chronic illness and an important cause of avoidable mortality. The prevalence in the North East Region of England is estimated at 5.6% (http://www.yhpho.org.uk/resource/view.aspx?RID=81090) equating to approximately 96 patients per full time general practitioner. A review of quality of care studies (including diabetes) in UK primary care concluded that ‘in almost all studies the process of care did not reach the standards set out in national guidelines or set by the researchers themselves’ [1]. A national audit of diabetes care showed that less than 50% of patients received all nine key aspects of diabetes care, with considerable variability across the country [2]. However, care for people with diabetes has been improving: data from the UK’s voluntary incentive system for promoting high-quality care—the Quality and Outcomes Framework (QOF)—shows that quality scores across the UK are high, though many indicators are arguably relatively undemanding. However, for the most demanding indicators (*e.g.*, relating to tighter levels of HbA1c or blood pressure control) QOF performance is lower. Some of this variability likely reflects patient physiology or behaviour, but also reflects variable clinician management.

Recognition of quality gaps has led to increased interest in implementation research (the scientific study of methods to promote the systematic uptake of research findings into routine clinical practice) over the past 15 years [3,4]. The transfer of research findings into practice is often slow, leading to gaps in quality of care [5]. Interventions to improve the quality of care that patients receive can be effective, but previous studies provided little theoretical or conceptual rationale for their choice of intervention [6] and only limited descriptions of the interventions and contextual data [7]. We have argued [8] that a poor understanding of potential barriers and enablers to implementation limits the capacity to design appropriately targeted interventions. The challenge for implementation researchers is to develop and evaluate a theory-based approach to intervention design that will offer a generalisable framework for research and support the choice and development of interventions. The present cluster randomised controlled trial represents a response to this challenge, building upon recently completed development work identifying theoretical constructs that consistently predict healthcare professional behaviour and that are potentially modifiable in an intervention [9,10].

### Evidence base for intervention development to identify theory: the improving quality in diabetes (iQuaD) study

Results from our recently completed study directly informs this protocol [9,11]. The ‘improving Quality in Diabetes’ (iQuaD) study was a national, theory-based study of the structural, organisational and individual factors associated with the performance of six healthcare professional behaviours involved in managing type 2 diabetes in primary care in the UK. We identified six clinical behaviours covering a range of clinical activities (prescribing, non-prescribing), some of which were challenging (*e.g.*, controlling blood pressure and HbA1c that was above target despite other drug treatment), and reflected recommended best practice as described by national guidelines at the time. Data were collected by telephone interview to a practice study contact, postal questionnaire to practice staff, postal questionnaire to patients, and from patient records. Ninety-nine practices completed a telephone interview and responded to baseline questionnaires assessing constructs from a range of theories. Response rates for all surveys and all professional groups were >75%. Scores on beliefs about the six target behaviours were generally consistent with good practice [9], indicating that care gaps are likely a function of difficulties in regulating clinical behaviour in context, rather than knowledge deficits. We also showed that there is sometimes a discrepancy between what clinicians report providing and what patients report receiving [12].

We assessed practice attributes and a wide range of individually reported measures at baseline; measured clinical outcomes over the ensuing 12 months, and administered a number of measures of clinical behaviour at baseline and at 12 months. A principal finding of iQuaD was that there continues to be variability in these clinical behaviours and most clinical behaviours that we investigated were not being optimally performed (see Table 1).

**Table 1:** Targeted clinical behaviours and evidence of performance from the iQuaD study, and associated NICE quality standards

**iQuaD clinical behaviour targeted**	**Behavioural performance from iQuaD**	**NICE quality standard**^**a**^
**1.** Over the past 12 months **provided general education** about diabetes for patients with type 2 diabetes	Received by 73% of patients (via patient survey)	QS1 - ‘People with diabetes and/or their carers receive a structured educational programme that fulfils the nationally agreed criteria from the time of diagnosis, with annual review and access to ongoing education’
**2.** Over the past 12 months **provided advice about weight management** to patients with type 2 diabetes whose BMI is above a target of 30 kg/m2 even following previous management	Received by 51% of people whose BMI was above a target of 30 kg/m2, even following previous management (via patient survey)	QS2 - ‘People with diabetes receive personalised advice on nutrition and physical activity from an appropriately trained healthcare professional or as part of a structured educational programme’
**3.** Over the past 12 months **provided advice about self-management** for patients with type 2 diabetes	Received by 68% of patients (via patient survey)	QS3 - ‘People with diabetes participate in annual care planning which leads to documented agreed goals and an action plan’
**4.** Over the past 12 months **prescribed additional therapy for the management of glycaemic** control (HbA1c) in patients with type 2 diabetes whose HbA1c is higher than 64 mmol/mol (8.0%) despite maximum dosage of two oral hypoglycaemic drugs	Received by 59% of people whose HbA1c was higher than 8.0%, despite maximum dosage of two oral hypoglycaemic drugs (via practice-held prescribing data)	QS4 - ‘People with diabetes agree with their healthcare professional a documented personalised HbA1c target, usually between 48 mmol/mol and 58 mmol/mol (6.5% and 7.5%), and receive an ongoing review of treatment to minimise hypoglycaemia’
		QS5 - ‘People with diabetes agree with their healthcare professional to start, review and stop medications to lower blood glucose, blood pressure and blood lipids in accordance with NICE guidance’
**5.** Over the past 12 months **prescribe additional antihypertensive** drugs for patients with type 2 diabetes whose blood pressure is 5 mmHg above target of 140 mmHg (systolic) or 80 mmHg (diastolic) even following previous management	Received by 40% whose blood pressure (BP) is above a target of 140 mm Hg for Systolic BP or 80 mm Hg for Diastolic BP, even following previous management (via practice-held prescribing data)	QS5 - ‘People with diabetes agree with their healthcare professional to start, review and stop medications to lower blood glucose, blood pressure and blood lipids in accordance with NICE guidance’
**6.** Over the past 12 months **examined foot** circulation and sensation in the feet of patients with type 2 diabetes	Received by 91% of patients (via patient survey)	QS10 - ‘People with diabetes at risk of foot ulceration receive regular review by a foot protection team in accordance with NICE guidance’
		QS11 - ‘People with diabetes with a foot problem requiring urgent medical attention are referred to and treated by a multidisciplinary foot care team within 24 hours’

^a^From NICE quality standard for diabetes in adults (http://www.nice.org.uk/media/7F8/B2/DiabetesQualityStandard.pdf).

Note. QOF = Quality and Outcomes Framework.

Our results showed that in primary care, individual healthcare professionals’ beliefs about these behaviours predicted their clinical behaviour while their perceptions about their workplace did not; multilevel models also showed that variability was primarily between individual clinicians within general practices rather than between the practices [10]. Based on these findings, an intervention targeting individuals within organisations rather than targeting their organisation may be more likely to be effective.

The present study is directly informed by considerable theoretical development work accomplished within the iQuaD study wherein we theorised and tested a model of healthcare professional behaviour that combined existing theoretical explanations of behaviour [13]. We initially tested ‘as originally theorised’ versions of multiple different individual [10], organisational [14] and stress theories [15], to investigate to what extent constructs from multiple theories predict six different behaviours in the same sample of health professionals (enabling direct comparison of findings across multiple behaviours). The research in iQuaD was itself based on previous theory-based research conducted with different samples of healthcare professionals [16-19]. In addition, we tested each theory using multilevel modelling to simultaneously account for individual and practice-level variability in theoretical constructs and clinical behaviour.

We showed particular constructs within particular behavioural theories to be consistently predictive of healthcare professional behaviour, including intention/proximal goals and self-efficacy (social cognitive theory [20]), post-intentional factors (action and coping planning [21]), and habit [22] (learning theory), theories which have a broader evidence base within psychology [23-25] and implementation science [26]. We tested organisational theories, including organisational justice, organisational citizenship, and team climate, and found that while organisational justice factors predicted some clinical behaviours cross-sectionally [14], none of the organisational-level theories consistently predicted clinical behaviours at 12 month follow-up [27].

We also hypothesised *a priori* how some of these theories might combine. We developed and operationalised a dual process model of health professional behaviour that simultaneously tested a sequential reflective process involving how intention to perform a clinical behaviour was mediated through post-intentional factors (action planning, coping planning) alongside a parallel impulsive process accounting for the degree to which clinicians behave automatically [13]. We demonstrated that, for most clinical actions tested, a dual process approach contributed to understanding how reflective and impulsive factors relate to clinical behaviours.

A separate literature has also investigated the role of multiple goal pursuit, investigating how clinicians [28,29] and other populations [30,31] manage competing and facilitating goals and priorities in the time available. This research demonstrated that goal conflict and facilitation are readily identified and predictive of clinical and health-related behaviours and will further inform the present intervention.

To drive forward further improvements in quality of care, the UK National Institute for Health and Care Excellence (NICE) published 13 Quality Standards (QS) for Type 2 diabetes that cover a broader range of areas of diabetes care than current QOF indicators. Eight of these directly relate to primary care and to clinical behaviours assessed in the iQuaD study, which suggests areas in which current care falls short of achieving these indicators. Table 1 describes the QSs that map onto the clinical behaviours that we have previously investigated and which will be targeted for change by the intervention described in this protocol, as well as rates of performance of each behaviour and the sources of data we have used to assess these outcomes.

iQuaD provided an unprecedented opportunity to test and develop further theory that could inform the design of an intervention to improve diabetes quality of care. On the basis of this theoretical development, we will develop, pilot, deliver, and evaluate a theory-based behaviour change intervention targeting GPs and practice nurses to promote high-quality diabetes care consistent with NICE quality standards.

### Aims

To conduct a cluster randomised controlled trial to evaluate the effectiveness and costs of a theory-based behaviour change intervention targeting general practitioners (GPs) and practice nurses/nurse practitioners, to support improvement in the provision of high-quality care for people with type 2 diabetes.

### Objectives

1. Recruit 44 general practices in the North East of England.

2. Develop the content and decide the method of delivery of a multi-faceted, targeted, theory-based intervention to change healthcare professional practice, targeting six key clinical behaviours that currently show room for improvement.

3. Deliver the intervention to GPs and nurses in 22 general practices.

4. Collect outcome data on the six behaviours by a combination of patient survey and computer data extraction 12 months after intervention delivery.

5. Collect process data on the six behaviours using semi-structured interviews (analysed qualitatively) and theory-based questionnaires (analysed quantitatively).

6. Assess the fidelity of delivery of the intervention.

7. Conduct a cost analysis using data on the cost of delivering the intervention and cost of healthcare resources used because of the intervention.

## Methods

### Trial design

The study will be a cluster randomised controlled trial with the general practice as the unit of randomisation and analysis to protect against contamination and given the level at which the primary outcome will be measured. The intervention will be a multi-level intervention, delivered to teams but targeting individual-level variables (see CONSORT checklist in Additional file 1).

### Participants

The primary study participants will be general practitioners (irrespective of status), practice nurses/nurse practitioners, and healthcare assistants working in the study practices actively engaged in providing diabetes care. To assess the clinical behaviour for processes of care that are not routinely recorded in patients’ notes, we will also recruit an anonymous random sample of 100 people with type 2 diabetes registered within each practice, asking them to report whether they have received particular aspects of care.

### Inclusion criteria

Practice level: any general practice providing care to people with type 2 diabetes in the North-East of England. Clinician level: any general medical practitioner, practice nurse/nurse practitioner or healthcare assistant involved in providing care to people with type 2 diabetes in the practice. Patient level: patients over 18 with type 2 diabetes registered with the practice.

### Exclusion criteria

Practice-level: practices included in pilot study. Clinician level: clinicians who work across multiple different general practices (*e.g.*, district nurses; to avoid contamination). Patient level exclusions: patients with type 1 diabetes or those for whom the primary care team consider it would be clinically inappropriate (*e.g.*, complex comorbidity, frailty, major psychiatric problems).

### Recruitment

#### Step one: general practice recruitment

Potential participating clusters (*i.e.*, general practices) will be invited to participate by research facilitators who will identify a practice contact as a primary point of contact for the research team. The study research associate (RA) will also present the study design at local GP research forums to garner interest. Once identified, the study RA will contact practices to obtain written practice-level agreement to participate in the trial in principle and to subsequently arrange details of attendance of GPs, nurses and healthcare assistants at the intervention delivery sessions.

#### Step two: clinician recruitment

Once practices are recruited, the study RA will contact practices to conduct a telephone interview to identify practice structure features and all clinicians in the practice providing care to people with Type 2 diabetes who may be interested in participating. Each identified clinician will be provided with a study invitation pack by post via the practice contact, including a letter of invitation, information sheet, consent form and baseline questionnaire.

#### Step three: patient recruitment

From our previous study [9], we identified that advising behaviours are not consistently recorded in patient notes. We therefore conducted a patient survey to ask patients to report on whether they had received this care in the past year. Consistent with this approach, we will invite a random sample of 100 patients with type 2 diabetes from each practice to complete an anonymous postal questionnaire 12 months after the delivery of the intervention. Patients with type 2 diabetes will be randomly selected from practice lists by the study contact in each recruited practice using a random sequence provided by the study statistician. Patients will be anonymous to the research team.

### Study setting

The study will be based in 44 general practices within the North East region of England.

### Intervention development

The development of the intervention will be consistent with MRC guidance for the development and evaluation of complex interventions and is an on-going process that began with our previous national study of theoretical predictors of diabetes care (iQuaD). iQuaD represents the Development phase of the MRC Framework, wherein we identified and developed theory and modelled process and outcomes. The present study will involve the feasibility and piloting domain and the evaluation domain of the MRC Framework, whilst continuing to involve the development process. The intervention will be piloted for feasibility and acceptability, and findings from the pilot will inform further refinement of the intervention as needed.

**Figure 1:**
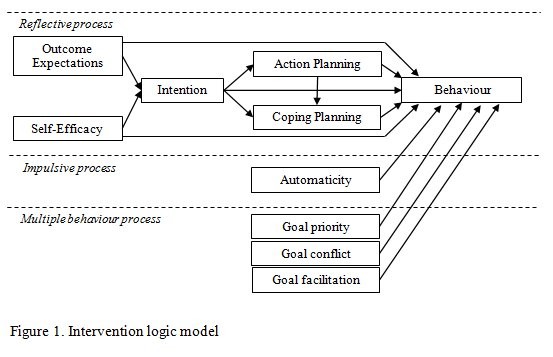
Intervention logic model.

The intervention content will be directly informed by theory-testing and theory-development results from iQuaD, where we demonstrated that motivational and action factors accounted for variability in clinical behaviour better than organisational factors [27]. Influenced by research demonstrating a sequential relationship between motivational and volitional factors and behaviour [32,33] and between reflective and impulsive processes [34,35], we also showed that clinical behaviour can often best be understood as the consequence of two processes operating in parallel; one rational, one habitual [13]. On the basis of these results, our intervention development will be informed by the logic model presented in Figure 1, which we have refined to focus on the key motivational and action factors shown to best explain clinical behaviour. This model will serve to guide the selection of recognised behaviour change techniques (BCTs [36]) to target the behaviour patterns we have found to be associated with lower rates of high-quality care in our previous study. We will also investigate the impact of the intervention on the priority of each behaviour, the extent that other clinical behaviours are perceived to facilitate or conflict with each target behaviour [28,29] and changes in time spent on each behaviour. We will aim to change the behaviours using an intervention (guided by the clinical content of evidence-based diabetes care) and we will investigate and pilot optimal methods of delivery tailored to suit the organisational structure of individual practices (*i.e.*, by whom, to whom and where). We will subsequently publish a full description of the intervention development and content.

### Control condition

The control group will be asked to complete baseline and follow-up process questionnaires, but will not receive any intervention session. The control group will not be able to access the intervention materials during the intervention period, but will be provided with access after the trial is complete.

### Intervention fidelity

An intervention fidelity study will be conducted to assess the validity and replicability of the intervention. This will cover the five standard elements of design, training, delivery (including coding BCTs used in the sessions), receipt, and enactment [37]. Delivery of the intervention will be assessed by analysing the transcripts of audiotapes of the full intervention for each session as well as via questionnaire-based facilitator report of delivery completed after each session.

### Primary outcomes

The primary outcomes will be based on behaviours as measured in our previous study, updated to be consistent with NICE Quality Standards for Type 2 diabetes. Updates include necessary minor modifications to the thresholds (compared to those used in our previous study) in the two prescribing behaviours (for HbA1c and blood pressure control) in light of evolving guidance, and the advising behaviours in light of the specific wording of the NICE Quality Standards. We will also explore the capacity to use QOF data as secondary sources of data, depending on the available indicators at the time of follow-up. Table 2 describes the primary outcomes, and the primary and secondary sources of data.

**Table 2:** Primary outcome measures and data sources

**Outcome (clinical behaviours)**	**Primary data source**	**Secondary data sources**
**1.Provide personalised nutrition advice** to patients with type 2 diabetes whose BMI is above a target of 30 kg/m^2^ even following previous management	Patient survey @ 12 months (receipt of advice)	Clinician survey @ 12 months; QOF
**2.Provide on-going education** about diabetes for patients with type 2 diabetes	Patient survey @ 12 months (receipt of education)	Clinician survey @ 12 months; QOF
**3.Provide personalised advice on physical activity** for patients with type 2 diabetes	Patient survey @ 12 months (receipt of advice)	Clinician survey @ 12 months; QOF
**4.Prescribe additional antihypertensive drugs** (*i.e.*, new drug or increased dosage) for patients with type 2 diabetes with uncomplicated hypertension whose blood pressure is at least 5 mmHg above 140/80 mmHg even following previous management	Practice computer prescribing data @ 12 months	Clinician survey @ 12 months; patient survey @ 12 months; QOF
**5.Prescribe additional therapy for the management of glycaemic control (HbA1c)** in patients with type 2 diabetes whose HbA1c is higher than 58 mmol/mol (7.5%) despite maximum dosage on all oral hypoglycaemic drugs	Practice computer prescribing data @ 12 months	Clinician survey @ 12 months; patient survey @ 12 months; QOF
**6.** Personally ensuring that circulation and sensation in the feet of people with type 2 diabetes have been examined in the past 12 months, by **examining feet yourself and/or referring** them	Practice computer management data @ 12 months	Clinician survey @ 12 months; patient survey@ 12 months; QOF

*Note.* QOF = Quality and Outcomes Framework.

### Process evaluation

#### Quantitative

To understand the intervention’s mechanism of change, we will survey participating clinicians in both groups prior to intervention delivery and again 12 months later to measure theoretical constructs targeted by our intervention and changes in these constructs associated with the intervention [38]. We will assess constructs based on models and measures from the iQuaD study including constructs from social cognitive theory (intention, self-efficacy, outcome expectancies), volitional constructs (action planning, coping planning) and behavioural automaticity for each of the six targeted clinical behaviours. In addition, we will also investigate whether the intervention alters perceptions of goal facilitation, goal conflict and goal priority between clinical behaviours based on recently developed short measures [29], as well as measures of clinicians’ time usage. Finally, given the team-based nature of primary care diabetes management we will explore whether the intervention alters any perceptions of team climate (participation, support for innovation, vision, and task orientation).

#### Qualitative

There will be two qualitative components to the study. First, a preliminary set of interviews will be conducted with a convenience sample of GPs and nurses to assess perceived barriers and facilitators to engaging in each of the six target behaviours. The results of this work will help to inform the development of the quantitative questionnaires. The interviews will also involve assessing preferences for how interventions might be delivered in a feasible and accommodating way and findings will serve to further inform development of intervention content and delivery. Second, after the delivery of the intervention we will conduct an interview study with GPs and nurses in a sample of four practices receiving the intervention to assess perceived intrapersonal, interpersonal, contextual barriers and facilitators to engaging with the intervention sessions themselves, subsequent perceived changes in practice, and usage of the intervention materials. These interviews will also serve to inform the intervention fidelity assessment. Given that interviews involve a time burden and may constitute a co-intervention involving prompting, we will conduct the interviews after the follow-up outcome data has been collected.

### Economic evaluation

The economic evaluation will comprise a cost analysis. All costs will be expressed in 2012/2013 values. The costs of delivering the intervention will be collected. Two main sources will be used to assign costs to healthcare resources [39,40] supplemented when necessary with unit cost data from other official sources and local surveys. Drug costs will be taken from the British National Formulary [41]. Within the trial a questionnaire at 12-month follow-up of an anonymous random sample of 100 patients from each practice will be asked to report on the use of NHS (National Health Service) services and medications over a twelve-month period. No discounting will be applied. A simplifying assumption will be made that the use of all costs and resources occurred at the beginning of the period. Specifically, the economic evaluation will involve the following:

1. Assessment of the costs of delivering the intervention in terms of staff training costs (both facilitator costs and GP time used in training) and any increase in the amount of standard patient education leaflets used by practices.

2. The average cost per patient to the NHS of medications prescribed in relation to hypertension, glycaemic control, and weight control. Conditional on available data, typical medication costs/usage across the intervention group will be compared to the general population. Differences between control and intervention groups will also be explored.

3. Costs of service usage: using self-reported questionnaires at 12 months from an anonymous sample of patients with type 2 diabetes from each participating practice, we will seek information on the use and NHS cost of community based weight control interventions. We will also seek to use practice records to assess the annual use of diabetes related services within practices. We will explore the use of comparator data that would allow us to forecast the additional workload that a practice would face as a result of successfully changing clinical behaviour and any induced change in patient uptake of practice based diabetes related services. Intervention and control groups will be compared, additionally national level data on service use by diabetes patients will be utilised for an additional comparator group.

### Sample size

Practice-level: 44 practices. Individual-level: The study is powered to detect change in the primary outcomes (six different clinical behaviours) between intervention and control practices. For advising behaviours, the primary outcome measure will be patients’ reports of having received the specific behaviours. In this respect, a random sample of 100 patients with type 2 diabetes in each practice will be surveyed. For prescribing and examining behaviours, data will be extracted from the patient computer records of all patients with type 2 diabetes in each practice. Based on our previous study an intra-cluster correlation coefficient (ICC) of 0.06 is expected and to detect a 15% absolute improvement in any of our measures of behaviour (conservatively calculated as 42.5% to 57.5%) with 80% power and 5% significance, our primary and conservative sample size estimate is that we will need 20 patients from a total of 40 practices. As this sample size does not account for any potential loss to follow-up, we will recruit a further four practices (44 in total). Accordingly, two groups of 22 practices will provide us with 83.9% power to detect a 15% improvement in any behavioural outcome. Our previous study (iQuaD) has demonstrated that this level of recruitment at the patient level is achievable, as we received postal outcome data from a mean of 41 patients per practice (having invited 100) and extracted prescribing data on all patients with type 2 diabetes in the practice (from analysing these data we identified that about 20 patients per practice were eligible for the specified prescribing behaviours). Based on our previous study we anticipate a mean sample size of 40 patients per practice and will thus have greater power. Assuming 40 practices (two groups of 20), we would have 89% power to detect a difference of 15 percentage points (42.5% versus 57.5%) assuming a type 1 error rate of 5% and an ICC of 0.06; retaining all 44 practices would increase power to 92%.

### Interim analyses and stopping guidelines

There will be no interim analysis for the primary trial outcomes. We will analyse baseline process evaluation data from clinician questionnaires after the intervention has been delivered. This will include descriptive statistics, psychometric properties of measures, and exploration of the association between process variables as a preliminary test of the theorised process models. This will be conducted by the study statistician without knowledge of group allocation.

The intervention aims to promote optimal performance of clinical behaviours that are recommended in policy documents and clinical practice guidelines and thus represents a very low risk. Therefore, we have no explicit stopping rules.

### Randomisation: sequence generation

The unit of randomisation will be the general practice: Randomisation will be performed by a statistician independent of the study team, using computer-generated random permuted blocks and will be stratified by practice size (the only organisation characteristic found to be related to performance in our previous study, iQuaD).

Random sample of 100 patients within clusters: The study statistician will provide each practice with a computer-generated list of random numbers to use for selecting which patients with type 2 diabetes to send a survey from their practice diabetes register.

### Randomisation: type

Permuted-block randomisation, with practice list size as the blocking factor; 22 practices will be randomised to intervention, and 22 to control. The size of the permuted blocks will vary randomly.

### Randomisation: allocation concealment mechanism

Each practice will eventually become aware of their allocation but allocation will be concealed from them until baseline process evaluation questionnaires have been returned to the research team. Within a practice, patients are not being allocated to alternative treatments so allocation concealment is not an issue.

### Randomisation: implementation

Clusters: NHS North of England Commissioning Support Unit will be involved in inviting all clusters to participate prior to randomisation. The study RA will send the study statistician anonymised practice ID codes and associated patient list sizes. The study statistician will generate the random sequence and allocate practices to two separate dummy-coded groups without knowledge of which group will receive the intervention; the study statistician will remain blind to allocation until follow-up data has been collected. Random allocation of clusters to intervention or control will take place consecutively as practices are recruited and until our target sample size (44) is reached.

Patients: The study statistician will generate the random sequence but will not be involved in the implementation of the randomisation. The statistician will provide each random number list to the study RA, who will send a random number list to the practice contact at each of the 44 practices to be used to select a random sample of 100 patients per practice to be sent questionnaires.

### Blinding

Given the nature of the intervention, GPs, nurses and healthcare assistants will inevitably be aware of their allocation and thus blinding of participants will not be possible. Each practice contact will become aware of allocation. To minimise recruitment bias, random allocation to intervention and control will take place after practices are recruited but practices will not be informed of allocation until after both practices and individual clinicians are identified and recruited (*i.e.*, not until after baseline clinician questionnaires are returned completed). Patients within each practice will remain blind to allocation. The outcome assessor for data from the practice computers will be kept blind to the allocation. The practice contact that will use the random sequence of numbers to identify the random sample of 100 patients within each practice invited to complete the patient survey will possibly not be blind to allocation. Blinding of the full research team is also not possible as intervention practices will be contacted to arrange the intervention sessions. However, the study statistician conducting the outcome analysis will remain blinded to allocation until after the outcome data have been collected at follow-up.

### Materials and procedures

Through the conduct of our previous study (iQuaD) we have developed and tested many of the instruments and data collection methods that will be used in the trial described in this protocol. The study outcome measures will be the proportion of patients recorded or reporting appropriately receiving each of the six study behaviours. We have successfully measured these behaviours in our previous study [9].

### Outcome measures (patient survey)

We will use a short questionnaire to assess patients’ reports of having received care, assessed 12 months after the intervention delivery. The three advising behaviours are not reliably recorded in general practice computer systems [9], and measures of these will be acquired by a self-administered patient survey.

Using an anonymous survey we will ask people with diabetes in the study practices about their experiences of their clinicians providing advice about nutrition, physical activity, general education about their diabetes and foot examination, and their confidence in understanding and enacting of their diabetes care.

Each practice will be provided with 100 patient questionnaires, coded by the research team with their practice study code and a patient code of 1 – 100 (*e.g.* 001/001), and 100 reply paid envelopes. Patients will return their questionnaires directly to the research team. The practice will not be aware of which patients have or have not responded to the questionnaire, giving further protection to patients’ confidentiality.

We will explore the potential of conducting a second mailing to the same random sample of patients a month later to promote a higher response. Patient consent to participate in the anonymised survey will be assumed by the receipt of a completed questionnaire.

### Outcome measures (computer read codes from patient electronic records)

#### Prescribing behaviours and foot examination

The behaviours relating to prescribing (prescribing additional antihypertensive drugs, prescribing additional therapy for managing glycaemic control) and foot examination are measured by extracting clinical and prescribing data from general practice computers using electronic queries developed and used in our previous study. We will study both the performance of clinical behaviours (measuring HbA1c, BP, foot checks) and the associated biochemical/physiological measurement (level of HbA1c; level of BP), accepting that there will be variability in the latter measures reflecting patient physiology and health behaviour. The data will relate to the 12-month period following intervention delivery and be extracted after month 12. Data will be extracted for a 24-month period (*i.e.*, 12 months prior to and 12 months after the month within which the intervention is delivered). We will thus account for any seasonal effects in performance of the target behaviours. The search queries will be sent to each practice, with written guidelines for how to run the query. For all registered patients with diabetes the research nurses will run computer queries to extract data on the total number of patients with diabetes in the practice and the number who have had: a foot check; blood pressure, HbA1c, and weight measured; level of systolic and diastolic blood pressure, level of HbA1c, cholesterol and body mass index; and diabetes-related medication (hypoglycaemic drugs, weight reducing drugs). We will pilot the data extraction procedure with an experienced NHS performance data manager to ensure reliability in data extraction.

#### Quality and outcomes framework data

The QOF is a voluntary annual reward and incentive programme for all primary care practices in the UK. The QOF details practice-level performance on a number of key indicators across organisational and clinical areas including diabetes. The data are publicly available from the NHS Information Centre http://www.ic.nhs.uk/. The QOF data for diabetes mellitus and practice organisation will be collected for each participating practice for the 12-month period of QOF data collection best matching the 12-month period before and again after baseline questionnaire completion.

### Process measures (clinician questionnaire)

We will post a baseline questionnaire prior to delivery of the intervention and a 12-month follow-up questionnaire to each identified practice contact for distribution to clinicians identified as involved in providing care to people with type 2 diabetes along with a postage paid return envelope. Two reminders will also be sent at baseline and follow-up to non-respondents to promote a higher response.

### Analysis

#### Main trial analysis

Practice attributes will be described. The primary analysis will involve testing, for each behaviour, whether there is a difference between intervention and control in the proportion of patients in each practice for whom the behaviour was undertaken during the 12 months following the intervention delivery. Rates of behaviour in intervention and control practices will be compared using a multilevel regression model taking into account the clustering of patients within practices. Practice list size will be included as a covariate given its use as a stratification factor. We will also consider specialist diabetes clinic and onsite insulin prescription as covariates depending on bivariate correlations. For the two prescribing behaviours the rates at which patients received the behaviour prior to the intervention will also be included as covariates in the analysis. The main analysis will be ‘intention to treat’; practices randomised to receive the intervention will be compared with other practices. A secondary ‘as treated’ analysis will take into account the uptake of the intervention in each practice.

In the case that outcome is missing for individual patients if we have other relevant variables for those individuals we will impute missing values using the ‘across cluster random-effects logistic regression method’ described by Ma *et al.*[42]. In the case of missing clusters no imputation will be undertaken. The potential impact of missing clusters on the estimated impact of the intervention will be assessed using sensitivity analysis involving a range of assumptions about the performance of the missing clusters.

#### Multiple comparisons

Each of the six individual behaviours will be analysed separately; for each behaviour, we will generate a 95% confidence interval for the effect of the intervention. The 95% confidence interval corresponds to a type 1 error rate of 5%. If we assume that the behaviours are independent, with six separate tests, the probability that one or more is significant at the five percent level is 0.265. Thus the effective type 1 error rate is 26.5% rather than the nominal 5% assumed in the power calculation. However, the probability of two or more tests being significant purely by chance is 0.033. In practice, the behaviours and associated tests are not independent but this figure can be used as a guide. Provided that the intervention is shown to have beneficial effects on at least two of the clinical behaviours this can be regarded as evidence that the proposed method can be used to develop effective interventions.

### Additional planned analyses

#### Secondary trial analysis

For the two prescribing behaviours, we will conduct an interrupted time series (ITS) analysis to investigate monthly changes over time between intervention and control practices, for 12 months prior to the intervention through 12 months post-intervention (accounting to timing of intervention delivery). We will supplement this analysis with relevant QOF data in each practice, using a non-equivalent dependent variables analysis to further bolster the ITS.

#### Exploratory analyses

To supplement the individual analyses, a pooled estimate of effect size will be calculated based on a random effects approach. A statistical model will be developed in which it is assumed that the size of the effect will not be the same for each behaviour but that the effect size varies randomly about some overall mean. Also, while the trial is designed and powered to detect changes in clinical behaviour, to inform future interventions, we will explore whether changes in clinical behaviour are related to changes in patient intention, self-efficacy and behaviour as well as changes in patient physiological outcomes (blood pressure, HbA1c).

#### Process evaluation analyses

Quantitative: We will use analytical methods we have previously developed to test for differences between groups on hypothesised targeted constructs, controlling for baseline differences [38]. Mediation models will be used to test whether intervention effects on behaviour are mediated through the targeted theoretical constructs, incorporating both sequential reflective and parallel impulsive processes.

Qualitative: Perceived intrapersonal, interpersonal, and contextual barriers and facilitators to engaging with the intervention will be coded using a coding framework and framework analysis informed by the Theoretical Domains Framework [43].

#### Fidelity analysis

Relevant portions of the recordings will be transcribed and analysed using a checklist of BCTs developed from the intervention protocol, to code whether the planned BCTs were actually delivered and whether the duration of delivery of each BCT changes over the course of the delivery period and between intervention facilitators. We will also distribute post-intervention feedback forms. Receipt of the intervention and enactment of targeted behaviours will be assessed through brief questionnaires delivered together with the post-intervention process evaluation questionnaire. We will also assess post-delivery facilitator debrief checklists of BCTs presumed to be delivered. Analyses will be descriptive and we will assess inter-rater reliability of BCT coding.

#### Cost analysis

A post trial economic analysis will estimate the costs of the intervention and cost of service use. Costs to populate the model will be based upon questionnaire responses from practices and patients and will be supplemented with secondary data such as the British National Formulary for medication costs where appropriate. Both probabilistic (to explore the impact of statistical imprecision) and deterministic (to explore other uncertainties and scenarios) will be estimated. Propensity score matching using parametric modelling techniques will be used to explore differences in average cost per patient of prescribing in relation to hypertension and glycaemic control between the intervention and control group.

## Discussion

This multi-site cluster trial reflects on-going development of an intervention consistent with UK Medical Research Council guidance on the development and evaluation of complex interventions. Despite policy initiatives such as the QOF, there remain gaps in care. The present intervention is novel in its acknowledgement and consideration of multiple behaviour change in clinicians, building on considerable development work. In so doing, it will address important and relevant questions concerning the need to move beyond an isolated focus on changing one behaviour towards a more comprehensive approach that seeks to not only change multiple behaviours, but also to understand the mechanisms underpinning multiple behaviour change. To achieve this, we propose a comprehensive evaluation including not only the main trial analysis focusing on primary endpoints, but also hypothesised pathways/mechanisms of action using behaviour change techniques; intervention fidelity to allow clearer understanding of whether interventions were consistently delivered as designed and if not, why not; theory-based quantitative process evaluation investigating change in targeted hypothesised pathways; qualitative investigation of barriers and facilitators to trial participation; and a cost analysis that will inform scalability (*e.g.* into continuing medical education). The fidelity study will be key to the interpretation of the trial findings and will also identify where intervention delivery can be improved if necessary. Such extended evaluation is consistent with forthcoming MRC guidance on process evaluation.

The study will capture a breadth of key recommended behaviours in diabetes management including prescribing, advising, and examining behaviours. While descriptively involving different types of activities, our intervention will involve targeting constructs that were consistent predictors across multiple clinical behaviours. We do not have any pre-conceived hypotheses concerning which or how many of the six targeted clinical behaviours will change, though we suggest that a positive trial will be defined as change in at least two of the six target behaviours. Of the six behaviours, foot examination was previously shown to be performed at a relatively high (though not optimal) rate and thus may be more challenging to show improvement, though we do still expect to change this behaviour.

The present intervention will focus on using a behaviour change approach to change the behaviour of individual clinicians within the practice to improve care and it will be delivered to primary care practices that will likely vary in their organisational structures. The trial will thus provide a test of the merit of a behavioural approach to changing practice. Overall, the trial will contribute substantive new knowledge to efforts to improve diabetes quality improvement efforts as well as new theoretical knowledge concerning the determinants of behaviour change and the effects of behaviour change techniques designed to change these determinants.

### Ethics approval

The study was approved by the London – Riverside National Research Ethics Service (NRES) Committee, research ethics committee reference number 12/LO/1742.

## Competing interests

JP, JMG, NS, MPE are on the *Implementation Science* editorial board but were not involved in the decision making process for this manuscript.

## Authors’ contributions

All authors contributed to the study design and to this protocol. MPE, JP, FFS, JJF, MJ, GH, JMG, MJ, ME, EK, MH, and MD contributed to obtaining funding and are named investigators on the grant. GH, TC, JMG and MPE contributed to the clinical aspects of the protocol. JP, FFS, JJF, MJ, JM, ME, EK, JMG, and MPE contributed to the behavioural science aspects of the trial protocol. NS contributed to the statistical aspects of the study. MH contributed to the health consumer aspects of the protocol. MD and HB developed the economic analysis. All authors contributed to drafting the manuscript and all have read and approved the final manuscript.

## Supplementary Material

Additional file 1 CONSORT 2010 checklist for IDEA protocol. Click here for file
